# Glial Heterotopia of the orbit: A rare presentation

**DOI:** 10.1186/1471-2415-11-34

**Published:** 2011-11-16

**Authors:** Ranju Sitaula, Gulshan B Shrestha, Nabin Paudel, Sneha Shrestha, Dev N Shah

**Affiliations:** 1 Department of Ophthalmology, B.P.Koirala Lions Centre for Ophthalmic Studies, Institute of Medicine, Tribhuvan University Teaching Hospital, Maharajgunj, Kathmandu, Nepal. Post Box No.-8750

## Abstract

**Background:**

Glial heterotopias are rare, benign, congenital, midline, non-teratomatous extracranial glial tissue. They may masquerade as encephalocoele or dermoid cyst and mostly present in nose. Herein, we present an unusual case of glial heterotopia of the orbit with unilateral blindness.

**Case presentation:**

A 6 year-old-boy presented with a progressive painless mass over the nose and medial aspect of the left eye noticed since birth. On examination, the globe was displaced laterally by a firm, regular, mobile, non-pulsatile and non-tender medial mass. The affected eye had profound loss of vision. Computed tomography scan showed a large hypodense mass in the extraconal space with no intracranial connectivity and bony erosion. The child underwent total surgical excision of the mass and histopathological examination confirmed glial heterotopia of the orbit.

**Conclusion:**

Though the incidence of this condition is rare, the need of appropriate diagnosis and management of such mass to prevent the visual and cosmetic deterioration is warranted. To our knowledge this is the first reported case of Glial heterotopia of orbit causing unilateral blindness.

## Background

Glial heterotopia also known as Nasal glioma represents collections of normal glial tissue in an abnormal location distant to the central nervous system with no intracranial connectivity [1]. The most common reported site of heterotopic neuroglial tissue is in and around the nose [2]. Sixty percent of gliomas are extranasal, 30% are intranasal, and 10% are both [3]. Although benign, they can cause significant local damage and cosmetic deformity by compressing and destroying the nasal cartilage and orbital wall. Gliomas form an uncompressible mass that does not increase in size on the valsalva testing and does not transilluminate. Unlike dermoids, they do not necessarily occur in the midline, or attach to sinuses or skin. Confirmatory diagnosis of Glial heterotopia can be done with the help of imaging and histopathological findings supported by immunohistochemical presence of glial fibrillary acidic protein and S100 protein [4]. Surgical excision is the mainstay of treatment for Nasal gliomas, but they must be differentiated from encephalocele and other midline nasal masses before the surgery is scheduled.

## Case Presentation

**Figure 1:**
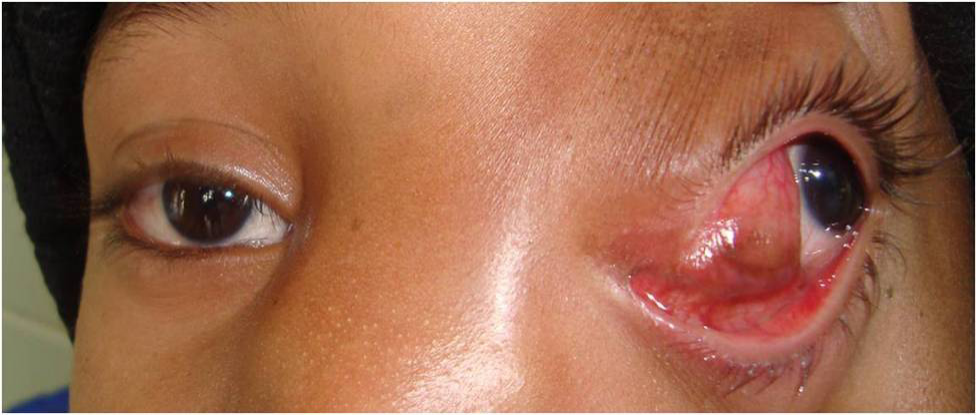
**Clinical photograph of mass in the medial aspect of left orbit causing left globe displacement**.

**Figure 2:**
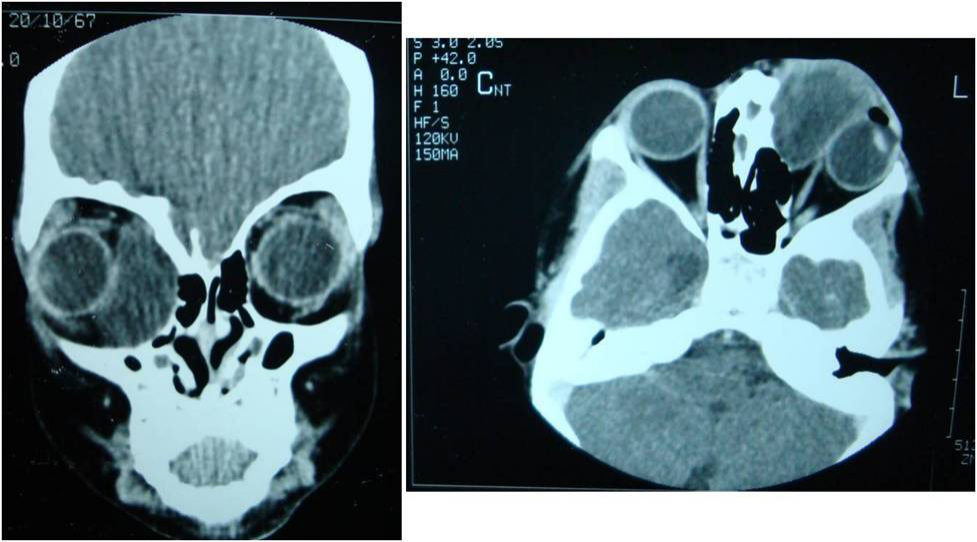
**Computed tomography of head and orbit showing a hypodense extraconal mass in the left orbit with no intracranial connectivity**.

**Figure 3:**
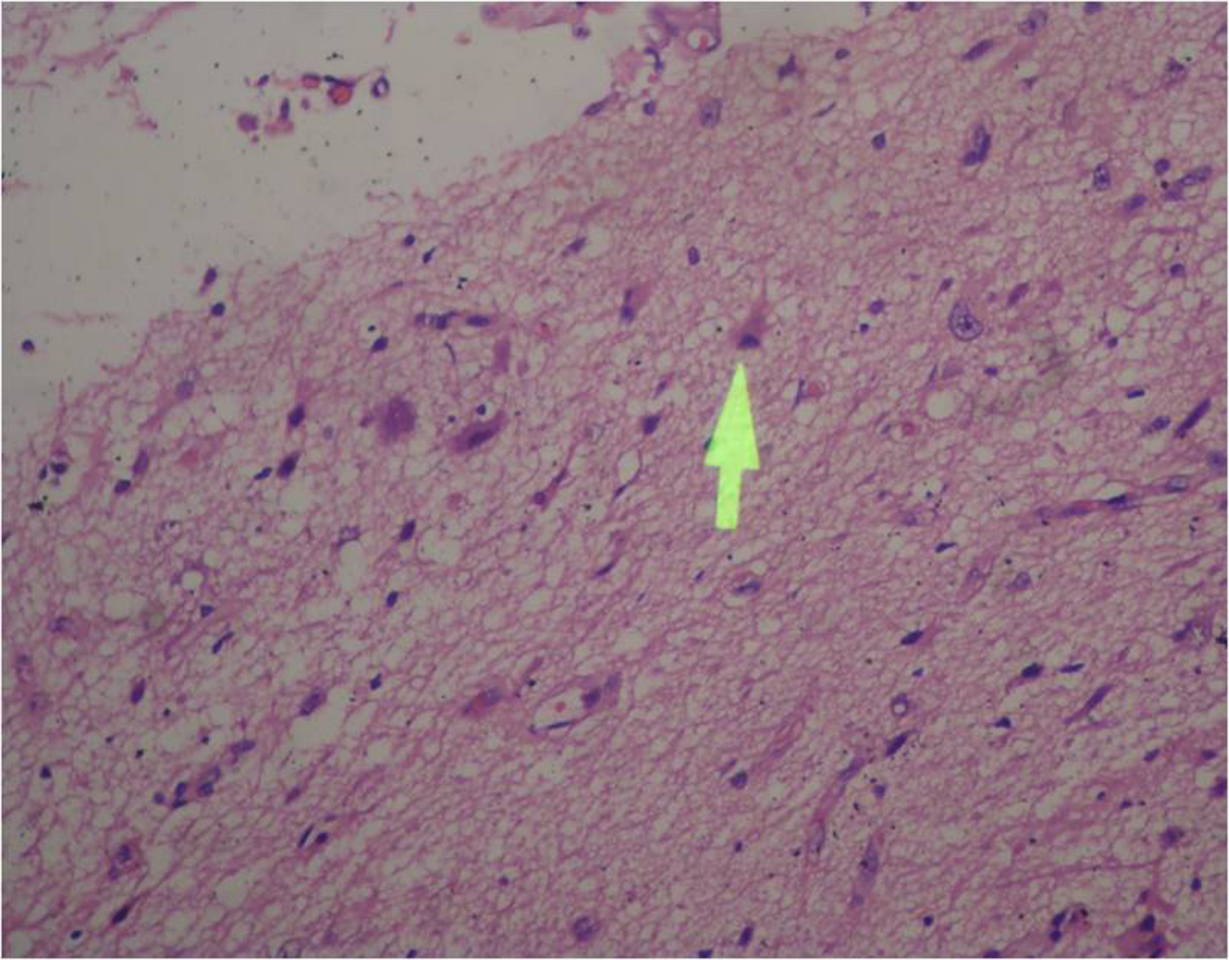
**Histopathological section showing well differentiated mature glial tissue surrounded by fibrous tissue and inflammatory cells**. The arrow showing astrocytes with fibrillary background. (Original magnification 200X) (the arrow was employed by the pathology department and due to lack of photography software you were unable delete or edit this.)

**Figure 4:**
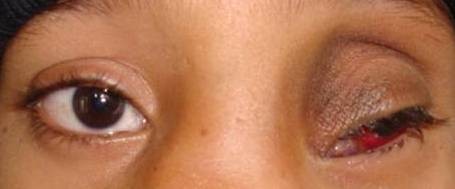
**Immediate postoperative clinical picture of the child**.

A 6-year-old boy presented to the outpatient department of our institution with complaint of a painless mass over the medial aspect of the left eye near the nasal bridge since birth. There was no history of pain, nasal bleeding or nasal obstruction. On examination, the best-corrected visual acuity was 20/20 in the right eye and 20/1200 in the left eye. There was a firm, regular, mobile, non-pulsatile and non- tender large mass involving the lateral part of nose and medial aspect of left orbit using lateral displacement of the left eye ball (Figure 1). The overlying conjunctival mucosal surface was keratinized. Corneal diameter as measured with vernier caliper was 11 mm each in horizontal and vertical meridian in right eye and 10 mm horizontally and 12 mm vertically in left eye. Anterior corneal radius of curvature of right eye was 7.22 mm (46.75D) vertically and 7.25 mm (46.5D) horizontally whereas in left eye it was 7.7 mm (43.75D) and 6.04 mm (56.0D) in vertical and horizontal meridian respectively. There were no other remarkable findings in the anterior segment. Axial length was 22.63 mm and 23.52 mm respectively in right and left eye. High compound myopic astigmatism (-0.50/-12.00X 090) was present in left eye whereas the right eye was emmetropic. There was no improvement in visual acuity of the affected eye even with best possible refractive correction. The posterior segment examination of both eyes was normal. Extraocular motility was full except restriction in adduction of left eye. Computed tomography scan showed a large (3.8 cm × 2.8 cm × 3.3 cm) hypodense globular lesion medial to left eyeball in the extraconal space with no direct connection to the brain (Figure 2). The mass had a thin enhancing wall with scalloping of the medial orbital wall without bony erosion. Sinonasal evaluation did not reveal the presence of significant sinus pathology except for the flat nasal bridge with medial displacement of lateral nasal wall. The patient underwent left transcaruncular medial orbitotomy under general anaesthesia and the mass was excised in total and was sent for biopsy. The histopathological examination revealed well-differentiated mature glial tissue composed of gemistocytic astrocytes and oligodendrocytes surrounded by fibrous tissue and focal inflammatory infiltrates (Figure 3). The wall fibrous tissue morphology predominates in long-standing cases and was present in our case. No meningeal or dural tissue was identified. Postoperatively there was no change in visual acuity but improvement in ocular motility including adduction. The postoperative cosmetic result was satisfactory (Figure 4). The child was followed 6 months after the initial presentation. There was significant improvement in ocular motility and regression in the corneal astigmatic power (-9.00X090) however the visual acuity remained the same. There was no sign of recurrence till that time.

## Conclusion

This case emphasizes on the appropriate diagnosis of the midline nasal masses and the importance of early diagnosis and treatment for the better visual and cosmetic prognosis. Earlier the diagnosis better is the treatment and the probability of visual recovery is more. They are generally present at birth, or become manifest within the first few years of life, but can be seen in any age group[1]. In our patient, though the mass was present since birth, the presentation was late due to illiteracy and financial barriers of the family.

Nasal gliomas may masquerade as encephalocoeles or dermoid cysts due to similar embryological origin. The nasal glioma, however, is ectopic sequestrated tissue and not a herniated structure[5] and careful examination is required to rule out their intracranial connection so that it may not lead to catastrophic CSF leaks during surgery[6].

To the best of our knowledge, the effect in visual and refractive status of the eye caused by these masses has been rarely addressed in previous reports. It is interesting to note that there was presence of -12.00D of against the rule astigmatism in our case. This signifies that the mass had been pressing the globe horizontally leading to the steeper horizontal meridian. The child was unilaterally blind most probably due to meridional amblyopia as suggested by the keratometry and refractive findings. Hence, the amblyogenic effect of the mass on the globe should also be considered while evaluating these types of cases.

## Consent

Written informed consent was obtained from the patient for publication of this case report and any accompanying images. A copy of the written consent is available for review by the Editor in-Chief of this journal.

## Competing interests

The authors declare that they have no competing interests'.

## Authors' contributions

RS and GBS were involved in medical and surgical management of the patient. RS, NP and SS were involved in literature review, conception, design and preparation of manuscript draft. DNS critically reviewed and provided important intellectual contents. All authors read and approved the final manuscript.

## Pre-publication history

The pre-publication history for this paper can be accessed here:

http://www.biomedcentral.com/1471-2415/11/34/prepub
